# Effect of COVID-19 Lockdown on Maternal and Infant Healthcare Services in an Urban Resettlement Area of East Delhi

**DOI:** 10.7759/cureus.52156

**Published:** 2024-01-12

**Authors:** Akansha Aggarwal, Khan A Maroof, Somdatta Patra, Dheeraj Shah

**Affiliations:** 1 Community Medicine, University College of Medical Sciences, Delhi, IND; 2 Pediatrics, University College of Medical Sciences, Delhi, IND

**Keywords:** healthcare-seeking behavior, breastfeeding, immunization, institutional delivery, child health, covid-19, home birth

## Abstract

Introduction: The coronavirus disease 2019 (COVID-19) pandemic has substantially disrupted essential maternal and infant healthcare services due to the diversion of resources. The imposition of lockdown was one of the critical strategies to flatten the curve in several countries, including India. This led to restricted access to pregnancy-related care, immunization services, and had an impact on home-based newborn care. We aimed to determine the effect of the COVID-19 lockdown on institutional deliveries and child healthcare services in a residential community of East Delhi.

Methods: This community-based, comparative study was conducted between January 2021 and August 2022. Seventy-seven families experiencing childbirth during the COVID-19 lockdown period (24^th^ March 2020 to 30^th^ November 2020) were compared with an equivalent number of families having childbirth during the corresponding period preceding the lockdown (24^th^ March 2019 to 30^th^ November 2019). The study involved face-to-face interviews conducted using a pretested and pre-validated interviewer-administered schedule.

Results: We found that non-institutional deliveries were substantially higher in the during-lockdown group (n=11, 14.3%) compared to the before-lockdown group (n=1, 1.3%) (OR=12.67 [1.59, 100.73]). Additionally, a significantly lower proportion of pregnant women received a minimum of four antenatal checkups (OR=8.26 [2.71, 25.23]), as well as iron and calcium supplementation during the lockdown. Reasons for non-institutional deliveries primarily included unavailability and denial of delivery services, as well as the fear of exposure to COVID-19 infection, as highlighted in our study.

A significantly lower proportion [OR=6.07 (2.56, 14.42)] of children were found to be immunized-for-age, along with a substantial delay in vaccination among those born during the lockdown period. There was a significant decrease in home visits by community health workers during both the antenatal and postnatal periods amidst the lockdown. Moreover, the proportion of children exclusively breastfed for six months was notably lower [OR=2.32 (1.17, 4.63)], and the age until which exclusive breastfeeding was continued was lower in the during-lockdown group.

Regarding healthcare-seeking behavior, services were sought by the families of 95.5% of children who fell sick during the lockdown period. Approximately 45.2% of families procured medicines from private health facilities, while about one-third acquired them from non-registered medical practitioners (NRMPs).

Conclusion: The COVID-19 lockdown significantly affected maternal and child healthcare services, leading to adverse outcomes across various crucial aspects. Institutional deliveries, antenatal care, community health worker visits, child immunization, and healthcare-seeking behavior were all adversely affected. In times of natural disasters like pandemics, it is crucial to establish specific provisions ensuring uninterrupted maternal and child healthcare throughout the lockdown. Integrating health education into essential services becomes imperative within the pandemic preparedness plan.

## Introduction

The availability and accessibility of antenatal, delivery, and postpartum care services, as well as infant care and immunization services, are crucial for ensuring the well-being and survival of both the mother and the child. Disruption at any step might lead to adverse outcomes [1]. Pregnant women opting for home delivery are at higher risk of maternal mortality due to complications like hemorrhage, eclampsia, sepsis, and obstructed labor.

The sustainable development goal (SDG) Target 3.1 aims to reduce the maternal mortality ratio (MMR) to less than 70 per 100,000 live births [2]. Globally and at the national level, significant progress has been achieved in successfully decreasing maternal and infant mortality so far. India has seen improvement in its MMR, dropping to 97 in 2018-20 from 113 in 2016-2018, and the current infant mortality rate (IMR) stands at 28 deaths per 1000 live births [3,4].

However, the emergence of the novel coronavirus disease 2019 (COVID-19) in December 2019 prompted various countries, including India, to implement full or partial lockdowns, impacting healthcare services and raising concerns about maternal and child health amidst the pandemic.

During this global crisis, concerns arose regarding the possibility of a significant number of women giving birth at home without proper assistance [5]. Furthermore, the lockdowns and the elevated risk of COVID-19 infection might have influenced the approach of pregnant women and mothers with newborns toward seeking healthcare services. They could have faced challenges in accessing services due to transport disruptions and lockdown measures or might have been reluctant to visit health facilities due to the fear of exposure to COVID-19 infection [5].

The mitigation strategies implemented to curb the COVID-19 pandemic might have also resulted in restricted access to child healthcare facilities, delayed vaccination schedules, and a decrease in home-based newborn care (HBNC) visits, especially in low- and middle-income countries [6].

The inadequate coverage of HBNC visits conducted by community health workers could have had an indirect effect on breastfeeding practices within society. We aimed to estimate the effect of the COVID-19 lockdown and disruption of healthcare services, on the availability and accessibility of maternal and infant healthcare services in an urban resettlement area of East Delhi. This article had been previously presented as a meeting abstract and poster at the 3rd Annual Conference of the Epidemiology Foundation of India, EFICON, AIIMS Patna, on November 4, 2022.

## Materials and methods

Between January 2021 and August 2022, we conducted a community-based comparative study in Nand Nagri, an urban resettlement area in East Delhi. Families where childbirth occurred during two specific periods were included for analysis: During the COVID-19 lockdown period (24th March 2020 to 30th November 2020) and, for comparative purposes, during the corresponding period before the COVID-19 pandemic (24th March 2019 to 30th November 2019).

For sample size estimation, we conducted a pilot survey involving 20 families in each group, i.e., during the COVID-19 lockdown period and the pre-COVID-19 lockdown period. The survey revealed a 25% difference in the proportions of institutional deliveries between the two groups, whereas the decline in institutional delivery as per the reported data of East Delhi was 5% [7]. Considering these figures, we concluded the estimated difference in institutional deliveries between the two groups to be 20% for the purpose of sample size calculation.

The sample size was estimated to be 77 in each group (N=77+77=154) at a 95% confidence level and 80% power for a two-tailed test. To achieve a sample of 154 deliveries, considering a birth rate of 15 per 1000 population per year in Delhi and an average of 6.5 family members per household in Nand Nagri, an estimated 1186 households were needed to be contacted. Multistage, universal sampling was used to select the participants.

Nand Nagri comprises five blocks housing a total of 21 sub-blocks, accommodating a population of 64,717 residing in 10,000 households [8]. On average, each sub-block contains approximately 500 households. To cover the required 1186 households, in the first stage, three blocks were randomly selected. In the second stage, one sub-block was randomly selected from each of these three selected blocks. Subsequently, households were approached consecutively within these selected sub-blocks until the desired sample size in each of the two groups was attained.

We conducted face-to-face interviews using a pretested and pre-validated interviewer-administered schedule. Our preference was for mothers to participate as respondents. However, in instances where the mother was unavailable, we interviewed a well-informed adult family member knowledgeable about maternal and infant healthcare. To obtain valid information about the delivery and child immunization, we cross-referenced information from institutional delivery documents and immunization cards available within the family. Verbal responses from the respondents were relied upon only in cases where records were absent or unavailable.

The schedule encompassed the following components: sociodemographic characteristics, pregnancy, and delivery-related information, specifics about infant immunization, infant feeding characteristics during the lockdown period and the pre-lockdown period, and child healthcare-seeking behavior during the lockdown period. In the schedule, there were certain closed-ended questions with multiple responses allowed. These questions pertained to reasons for non-institutional delivery, assistance during non-institutional delivery, and sources for obtaining medicines/drugs in the child healthcare-seeking behavior section.

Non-institutional deliveries comprised births that occurred at home, on the road, or outside a hospital setting. In instances of twin or triplet deliveries, one child was randomly selected and included in the study. The infant's immunization status was deemed delayed if any vaccination was postponed by seven or more days from the eligibility date for that particular vaccination.

The data were analyzed using SPSS 20.0 software (IBM Corp, Armonk, New York, USA). The chi-square test was used to find out the association between proportions of institutional delivery, immunized-for-age infants, and other relevant factors, while the student’s t-test was utilized for comparing means. Additionally, the Mann-Whitney U test was applied to assess continuous variables that exhibited non-normal distributions.

Prior to commencing the study, approval from the Institutional Ethics Committee-Human Research (IEC-HR) of the University College of Medical Sciences was obtained. The approval was granted under letter number IECHR/2020/PG/46/1-R1, dated 21st December 2020.

## Results

**Figure 1:**
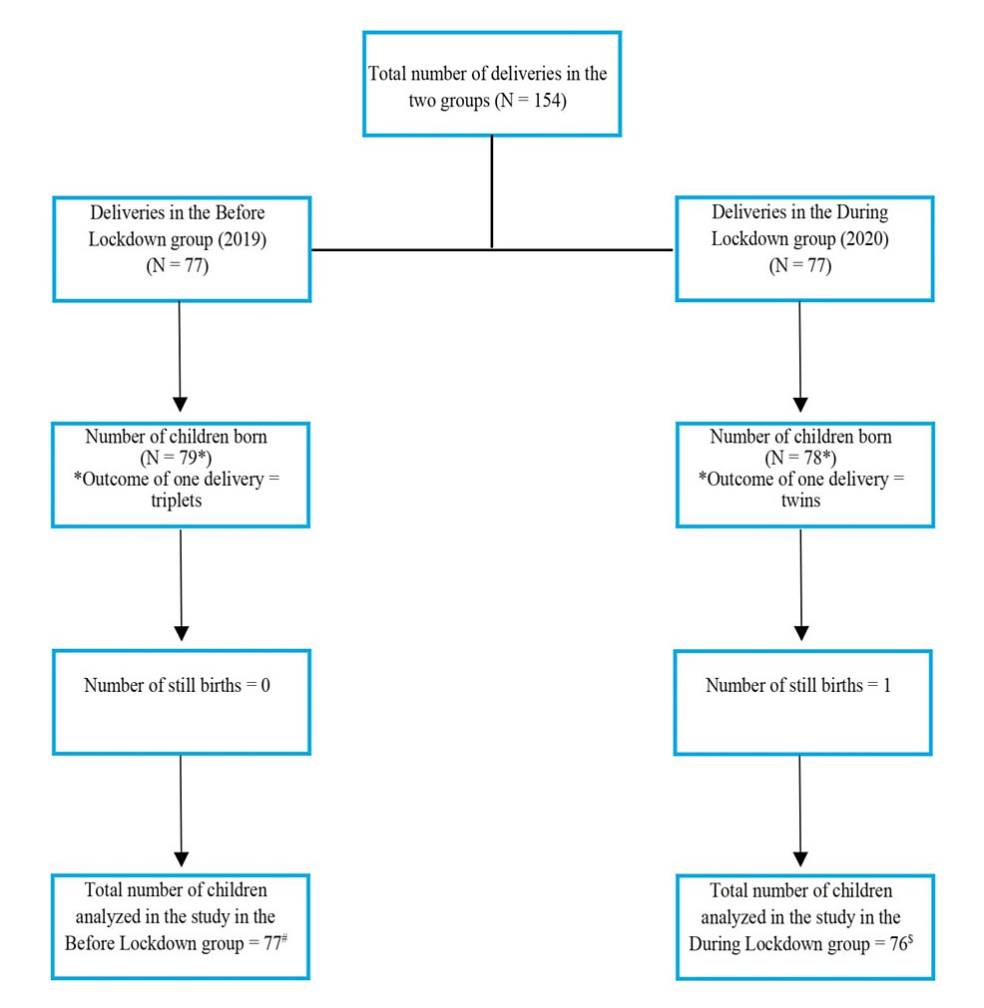
Flowchart depicting the number of deliveries, pregnancy outcome, and total number of children studied in the before COVID-19 lockdown group and the during COVID-19 lockdown group ^#^In Before-Lockdown group, in case of a triplet delivery, one child was randomly selected and included in our study. ^$^In During-Lockdown group, one stillbirth was excluded from the analysis of characteristics related to the children; and in case of a twin delivery, one child was randomly selected and included in our study.

The number of deliveries, pregnancy outcome, and total number of children studied in the before-COVID-19-lockdown group (BL group) and the during COVID-19-lockdown group (DL group) are shown in Figure 1.

We collected information regarding the 77 deliveries conducted during the COVID-19 lockdown period and the 77 deliveries conducted in the before-COVID-19-lockdown period. In the DL group, one mother had died during the postpartum period and therefore has been excluded from the analysis of characteristics related to the mothers. There was a triplet delivery and a twin delivery in the BL group and in the DL group, respectively. A single child was chosen from each of these and was included in the analysis randomly. There was one stillbirth in the DL group. Non-institutional deliveries were significantly higher (OR=12.67 [1.59, 100.73]) in the DL group (n=11, 14.3%) as compared to that in the BL group (n=1, 1.3%).

The sociodemographic characteristics of the families in the two groups are shown in Table 1.

**Table 1:** Sociodemographic characteristics of the families in the two groups ^#^N=76 as one mother in the During-Lockdown group had died during the postpartum period. *Statistically significant IQR: inter quartile range; Ref.: Reference category; SD: standard deviation

Sociodemographic characteristics	During-Lockdown group (N=77); n (%)	Before-Lockdown group (N=77); n (%)	P-value	OR (95% CI) (Ref.: Before-Lockdown group)
Mean (±SD) age of mothers (years)	28 (±3.5); (N=76^#^)	28.6 (±4.25)	0.34	-
Mothers’ education (High school education and above)	45 (59.2%); (N=76^#^)	40 (51.9%)	0.37	1.34 (0.71, 2.54)
Mothers’ occupation (Proportion of unemployed)	68 (89.5%); (N=76^#^)	62 (80.5%)	0.12	2.05 (0.81, 5.18)
Type of family (joint)	45 (58.4%)	37 (48.1%)	0.19	1.52 (0.80, 2.87)
Median (IQR) number of family members	6 (4, 7)	4 (4, 6)	0.01^*^	-
Proportion of families with upper socioeconomic class status (Revised Kuppuswamy scale)	43 (55.8%)	32 (41.6%)	0.08	1.78 (0.94, 3.37)
Median (IQR) family income	INR 15000 (10000, 20000)	INR 13000 (10000, 20000)	0.02^*^	-
Vehicle ownership status	53 (68.8%)	49 (63.6%)	0.50	1.26 (0.65, 2.46)
Median (IQR) number of children per mother	2 (1, 2)	2 (1, 2)	0.87	-
Respondent’s relationship with the child as a mother	66 (85%)	68 (88%)	0.63	0.79 (0.31, 2.04)

The antenatal services-related characteristics of the families in the two groups are given in Table 2.

**Table 2:** Antenatal service-related characteristics of the families in the two groups ^*^Statistically significant IQR: inter quartile range; ASHA: accredited social health activist; AWW: anganwadi Worker; Ref.: reference category

Antenatal service-related characteristics	During-Lockdown group (N=77); n (%)	Before-Lockdown group (N=77); n (%)	P-value	OR (95% CI) (Ref.: Before-Lockdown group)
Antenatal registration status (Not registered)	02 (2.6)	0 (0.0)	0.49	1.03 (0.99, 1.06)
Less than four antenatal checkups	24 (31.2)	04 (5.2)	<0.001^*^	8.26 (2.71, 25.23)
Tetanus and adult diphtheria (Td) vaccination status	76 (98.7)	76 (98.7)	-	1 (0.06, 16.28)
Not received IFA (iron folic acid) tablets	12 (15.6)	01 (1.3)	0.001^*^	14.03 (1.78, 110.84)
Not received calcium tablet	10 (13.0)	02 (2.6)	0.016^*^	5.59 (1.18, 26.46)
Median (IQR) number of home visits by ASHA/AWW during antenatal period	4 (2, 6)	6 (3, 8)	0.002^*^	-
Home visitation status by ASHA/ AWW during antenatal period (not visited)	07 (9.1)	06 (7.8)	0.77	1.18 (0.38, 3.69)

In the BL group, out of the total institutional deliveries (76), 6.6% (05/76) of pregnant women delivered at a private healthcare facility. Comparatively, in the DL group (66 institutional deliveries), this figure was notably higher at 24.2% (16/66) (P=0.003, OR=4.54 [1.56, 13.21]).

Regarding deliveries at public healthcare facilities, none of the pregnant women (0/71) in the BL group (71 deliveries at public healthcare facilities) delivered at a secondary healthcare facility. However, in the DL group (50 deliveries at public healthcare facilities), 12% (06/50) of women delivered at a secondary healthcare facility (P=0.004, OR=1.14 [1.03, 1.26]).

In the BL group, out of 72 pregnant women who had planned for an institutional delivery, only 1.4% (01/72) underwent a non-institutional delivery. However, in the DL group, 12.7% (09/71) of pregnant women who intended to have an institutional delivery ended up having a non-institutional delivery (P=0.02, OR=10.31 [1.27, 83.64]). In the DL group, 20.8% (16/77) of respondents reported difficulty in availing transportation during labor, while in the BL group, this was reported by 9.1% of participants (07/77) (P=0.04, OR=2.62 [1.01, 6.79]).

In the BL group, one female underwent non-institutional delivery whose out-of-pocket-expenditure (OOPE) was INR 600, whereas, in the DL group, 11 females had undergone non-institutional delivery, and the median (IQR) OOPE was INR 3000 (2000, 5000).

The reason(s) cited by respondents for non-institutional delivery (N=11) in the during-COVID-19 lockdown (DL) group were varied: delivery facility not available (36.4%, n=04); fear of exposure to COVID-19 infection (36.4%, n=04); denial of service at the facility (36.4%, n=04); delay due to referral (18.2%, n=02); facility was too far/ no transportation was available (18.2%, n=02); by choice (9.1%, n=01) and other reasons (27.3%, n=03) such as fear of denial of service at the hospital, lack of quality of care due to COVID-19 pandemic restrictions, excessive labor pain resulting in inability to go to the healthcare center, and short duration of labor pain. Two participants gave birth while either returning home or on their way to another hospital after being denied admission at a healthcare facility. One of these deliveries resulted in a stillbirth.

Among the 11 non-institutional deliveries in the DL group, nine were performed by a *dai* (traditional midwife), one was facilitated by a family member, and two were unattended. Additionally, three deliveries were assisted by other individuals, including an accredited social health activist (ASHA) worker, a homeopathy practitioner, and neighbors. Of the two unattended deliveries, one occurred in an auto-rickshaw, and the second took place outside the hospital's emergency ward after the hospital declined admission, resulting in a stillbirth.

The median (IQR) number of home visits conducted by community workers during the postnatal period was 3 (2, 5) visits in the BL group and 2 (0, 4) in the DL group (P=0.001).

In the DL group, there was one stillbirth, one early neonatal death occurring six days after birth, and one child aged less than 14 weeks. Consequently, the immunization history for vaccines recommended at 14 weeks has been documented for 74 children.

The study revealed that the odds of not being immunized-for-age with primary immunization vaccines during the COVID-19 lockdown was 6.07 times higher (95% CI= 2.56, 14.42) compared to the odds of not being immunized-for-age with primary immunization vaccines before the COVID-19 lockdown (41.3% versus 10.4%). Additionally, it was found that the number of children in the DL group who had received hepatitis B birth dose vaccine, OPV birth dose vaccine, and IPV 1st and 2nd dose vaccines was significantly lower compared to the children in the BL group (P<0.01). The delay for the Bacillus Calmette-Guérin (BCG) vaccine (P=0.004) and pentavalent/oral polio vaccine (OPV)/rotavirus 1st dose vaccines (P=0.03) was more pronounced among the children born in the DL group compared to those born in the BL group.

The details regarding breastfeeding and infant feeding characteristics in the two groups are presented in Table 3.

**Table 3:** Breastfeeding and infant feeding characteristics of the two groups ^*^Statistically significant IQR: inter quartile range; Ref.: reference category

Breastfeeding and infant feeding characteristics	During-Lockdown group; n (%)	Before-Lockdown group (N=77); n (%)	P-value	OR (95% CI) (Ref.: Before-Lockdown group)
Early initiation of breastfeeding (absent)	41 (53.90); (N=76)	36 (46.75)	0.37	1.33 (0.71, 2.52)
Exclusive breastfeeding for six months (infants> 6 months of age) (absent)	53 (73.6); (N=72)	42 (54.5)	0.016^*^	2.32 (1.17, 4.63)
Median (IQR) age till which Exclusive Breastfeeding was continued (months)	1.3 (0.03, 6.0); (N=73)	5.0 (2.0, 6.0)	<0.001^*^	-
Median (IQR) age at which complementary feeding was started (months)	6 (6, 6); (N=73)	6 (6, 6)	-	-
Proportion of infants given pre-lacteal feed after delivery	21 (27.6); (N=76)	13 (16.9)	0.11	1.88 (0.86, 4.1)

Out of the total 152 children who were available at the time of the interview, 132 (86.8%) suffered from at least one health-related problem during the COVID-19 lockdown period. Nearly half of them encountered respiratory problems such as cough, coryza, or breathlessness, along with experiencing fever. Additionally, around one-third of the children faced issues related to diarrhea.

Healthcare services were sought by the families of 126 (95.5%) children, with nearly half of the families (45.2%) acquiring medicines from private health facilities. Approximately 34.1% sought medication from non-registered medical practitioners (NRMPs), while roughly one-fifth (20.6%) obtained medicines from public health facilities. Moreover, 15.9% of families directly purchased medicines from local chemist shops.

## Discussion

Institutional deliveries dropped by approximately one-eighth in the DL group compared to the BL group. Other studies from Bangladesh, Nepal, and even our national data have reported a substantial decline [9-12]. The primary reason for not delivering in the hospitals was the fear of COVID-19 infection [1,13,14].

The disruptions in maternal and child health services due to COVID-19 can be attributed to various factors: limited mobility of patients and service providers, heightened fear of COVID-19 infection at healthcare facilities, financial obstacles to seeking services due to ongoing economic insecurity, shortages of safety equipment and healthcare personnel at the facilities. Additionally, challenges persisted in coordination between service providers and authorities to sustain regular services and ensure service preparedness [10].

Among the institutional deliveries, we observed a shift towards private facilities, accompanied by higher OOPE in the DL group. We found that there was a hike in the remuneration being charged by the traditional midwife during the lockdown period, aligning with findings from some studies, although contrary results exist [1,13,15]. The conversion of major public healthcare facilities into dedicated COVID-19 care centers compelled pregnant women to turn to private facilities for antenatal and delivery care services. Some opted for private facilities due to perceived poor quality of services and longer waiting times at public facilities, which according to them was worse during the COVID-19 lockdown. In Nepal, participants claimed that people had to resort to private healthcare services as an alternative option, which was more expensive and unaffordable for many of the vulnerable populations [16]. Additionally, the added expense of mandatory COVID-19 testing for all health facility visitors could have heightened OOPE.

We observed a decrease in the proportion of pregnant women receiving a minimum of four antenatal checkups during the lockdown period, a trend supported by other studies as well [1,10-11,13,15,17-18]. However, unlike our findings, some studies have reported a decline in antenatal registration [15,17]. Moreover, antenatal checkups were more frequent in private facilities.

There was a notable decline in antenatal and postnatal home visits, as well as in the distribution of iron and calcium supplementation during the lockdown period [18]. This observation aligns with data from the National Health Mission- Health Management Information System (NHM-HMIS), which showed a decrease in the number of newborns receiving the mandatory six visits from ASHA workers after institutional delivery, from 6,14,000 in June 2019 to 4,32,000 in June 2020 [7]. Similarly, for home deliveries, the count of women who received their first postnatal check-up within 48 hours of delivery decreased from 2,00,000 in June 2019 to 1,26,000 in June 2020 [18]. Another study reported a significant reduction in HBNC, with mothers stating the unavailability of ASHA and Anganwadi workers during the lockdown for antenatal or postnatal services [13].

We observed a significant reduction in the number of children immunized-for-age within the DL group. Similar findings were reported globally, nationally, and in other studies [1,14,18-20]. Across many Indian states, there was a noticeable decrease in all vaccine administrations, despite the fact that numerous hospitals remained non-COVID-19 facilities and maintained a continuous vaccine supply chain [1,14,20]. The decline in vaccination rates might be attributed to the diversion of ASHA and Anganwadi workers to tasks pivoted around control of the COVID-19 pandemic, such as conducting door-to-door surveys, awareness campaigns, overseeing the movement of migrants, and sensitization for social distancing. Furthermore, the decrease in outreach sessions, stringent lockdown measures, and disruptions in the vaccine supply chain could have also contributed to a dip in vaccination rates [21]. Even when vaccines were available at dispensaries, parents preferred to get their children vaccinated after the pandemic curve plateaued which led to a delay in vaccination.

We also noted a delay in vaccination for those vaccines that are given in the first few days of life, such as BCG, first dose of OPV, first dose of pentavalent vaccine, and rotavirus vaccine. Other studies have reported delays not only in these vaccines but also in most other vaccines during the lockdown period [22-24]. In our study, the delay in BCG vaccination within the DL group might be attributed to the increased proportion of non-institutional deliveries and deliveries in private facilities. Additionally, parents might have been hesitant to visit healthcare facilities with their newborns for vaccinations due to concerns about COVID-19 transmission.

In our study, we found that the number of infants of age ≥ six months with exclusive breastfeeding of ≥ six months was lower by 19% points in the DL group than in the BL group. A study conducted in Italy also reported a similar decline in exclusive breastfeeding among mother-baby dyads during the 2020 lockdown compared to mothers in 2018 [25]. Factors such as reduced visits from frontline workers during antenatal and postnatal periods, coupled with concerns about COVID-19 transmission through breastmilk likely contributed to the observed decline in exclusive breastfeeding rates.

Hospital containment measures adopted during the COVID-19 pandemic lockdown had a detrimental effect on maternal emotions and on breastfeeding exclusivity practices [13]. Surprisingly, a significant number of mothers expressed beliefs that breastfeeding should not be continued if the mother has received the COVID-19 vaccine, contrary to evidence-based recommendations [26]. This perception could have played a major role in the decrease in exclusive breastfeeding in the DL group. In contrast, a study in the United Kingdom revealed that most mothers were willing to continue breastfeeding even if they tested positive for COVID-19 [27]. These differences in maternal opinions could stem from varying levels of awareness about the transmission routes of COVID-19 infection.

We found that during the COVID-19 lockdown, over half of the families sought healthcare services from private facilities. The WHO highlighted that essential services for sick children were partially disrupted in approximately half of the world's countries during the pandemic [28]. Families encountered numerous challenges in accessing healthcare: fear of contracting COVID-19 at hospitals, financial constraints, especially when they had to avail the service from private healthcare facility, unavailability of services, transportation limitations, and service denial, leading to multiple visits to healthcare facilities or reliance on NRMPs. In Nepal, a qualitative study revealed that the increasing stigma surrounding COVID-19 discouraged early healthcare-seeking behavior, fostering a tendency to conceal the disease [16].

A potential limitation of our study might be the recall bias, particularly among participants from the BL group. We tried our best to mitigate this by conducting face-to-face interviews and cross-checking the responses with available records and follow-up questions.

## Conclusions

The study results revealed a significant decline in the number of institutional deliveries and the utilization of antenatal and postnatal services during the COVID-19 lockdown period as compared to the corresponding period before the lockdown. Additionally, the lockdown had a negative impact on the proportion of exclusively breastfed children and immunized-for-age children with an increase in the delay in vaccination, and it also affected the child healthcare-seeking behavior. Families encountered multiple barriers in seeking maternal and child healthcare services during the lockdown.
